# Continuous detection and genetic diversity of human rotavirus A in sewage in eastern China, 2013–2014

**DOI:** 10.1186/s12985-016-0609-0

**Published:** 2016-09-13

**Authors:** Nan Zhou, Dong Lv, Suting Wang, Xiaojuan Lin, Zhenwang Bi, Haiyan Wang, Pei Wang, Huaning Zhang, Zexin Tao, Peibin Hou, Yanyan Song, Aiqiang Xu

**Affiliations:** 1 School of Public Health, Shandong University, Jinan, 250012 China; 2 The 456th Hospital of PLA, Jinan, 250031 China; 3 Academy of Preventive Medicine, Shandong University, Jinan, 250014 China; 4 Shandong Provincial Key Laboratory of Infectious Disease Control and Prevention, Shandong Center for Disease Control and Prevention, No. 16992, Jingshi Road, Jinan, 250014 People’s Republic of China

**Keywords:** Rotavirus, Molecular epidemiology, Quantification, Sewage, China

## Abstract

**Background:**

Rotavirus is the leading viral agent for pediatric gastroenteritis. However, the case-based surveillance for rotavirus is limited in China, and its circulation in the environment is not well investigated.

**Methods:**

From 2013 to 2014, rotavirus was detected in raw sewage samples of Jinan and Linyi by quantitative PCR (qPCR) and conventional reverse transcription PCR (RT-PCR). After sequenced and genotyped, sequences analysis was conducted.

**Results:**

A total of 46 sewage samples were collected monthly for the detection of rotavirus, and rotavirus was positive in 43 samples (93.5 %, 43/46). By quantitative assessment, the concentrations of rotavirus in raw sewage ranged from 4.1 × 10^3^ to 1.3 × 10^6^ genome copies (GC)/L in Jinan, and from 1.5 × 10^3^ to 3.0 × 10^5^ GC/L in Linyi. A total of 318 sequences of 5 G-genotypes and 318 sequences of 5 P-genotypes were obtained. G9 (91.8 %, 292/318) and P[8] (56.0 %, 178/318) were the most common G- and P-genotype, respectively. Multiple transmission lineages were recognized in these genotypes. Interestingly, an intragenic recombination event between two G9 lineages was observed.

**Conclusions:**

This study provided the first report of comprehensive environmental surveillance for rotavirus in China. The results suggest that the concentration of rotavirus in raw sewage was high, and multiple rotavirus transmission lineages continuously co-circulated in Shandong.

## Background

Rotavirus is the leading viral agent attributable to pediatric acute gastroenteritis, and belongs to genus *Rotavirus*, family *Reoviridae*. Its genome contains 11 double-stranded RNA (dsRNA) segments, encoding six structural proteins (VPs) and five (or six) nonstructural proteins (NSPs). Rotavirus is divided into seven groups, and group A, B and C can infect humans. Of these, rotavirus group A (RVA) is predominant, which contains at least 27 G- and 37 P- genotypes based on the VP7 and VP4 sequences [1]. It was estimated that rotavirus infections led to about 0.45 million deaths in children younger than 5 years in 2008 globally, accounting for 37 % of deaths caused by diarrhea [2]. Thus, long-term surveillance of rotavirus is important.

Currently, rotavirus surveillance was mostly carried out in clinical cases. However, asymptomatic rotavirus infection is common [3], and case-based surveillance can only focus on the symptomatic cases. The epidemiology of rotavirus in asymptomatic individuals was not well investigated. Because rotavirus particles can be excreted to urban sewage system from both symptomatic and asymptomatic individuals, we can obtain more comprehensive molecular characterizations of rotavirus by sewage analysis. Previous studies have proved the feasibility and effectiveness of this novel approach for rotavirus surveillance [4, 5]. In addition, the quantification of rotavirus based on quantitative PCR (qPCR) in sewage has been conducted to better evaluate the seasonal profile of rotavirus and perform risk assessment [4, 6].

In China, rotavirus was the most frequently detected pathogen among children with diarrhea [7]. G3, G1, P[8] and P[4] were the most prevalent G- and P-genotypes [8]. Though the Lanzhou Lamb rotavirus (LLR) vaccine consisting of the monovalent serotype G10P[12] has been licensed since 2001, it was not included in China’s Expanded Program on Immunization. There were still more than 50,000 deaths of rotavirus-specific children under the age of 5 years from 2003 to 2012 in China [9]. Moreover, in China, case-based rotavirus surveillance is limited, and environmental surveillance for rotavirus is rare. The concentration of rotavirus in raw sewage is still unknown. In view of these, based on the successful practices of sewage surveillance for other enteric viruses in China since 2008 [10–13], we detected rotavirus in raw sewage of Jinan and Linyi in Shandong Province from 2013 to 2014, to obtain the epidemiological characterizations of rotavirus circulating in the local population and quantitatively assess the rotavirus in raw sewage in eastern China.

## Methods

### Sampling

In 2013 and 2014, raw sewage samples were collected monthly in the inlet collector canal of wastewater treatment plants (WWTPs) in Jinan and Linyi of Shandong Province, eastern China, with metropolitan populations of 2.6 million and 1.9 million, respectively. Each sample was transported, stored at a cold temperature (4 °C) and processed within 24 h.

### Virus concentration

An adsorption-elution method was used to concentrate viruses from sewage samples as described previously [14]. Briefly, sewage sample (1 L) was centrifuged at 3000 × *g* for 30 min at 4 °C. MgCl_2_ (2.5 mol l^−1^) was added to the supernatant to a final concentration of 0.05 mol l^−1^, and the pH was adjusted to 3.5 by hydrochloric acid (0.5 mol l^−1^). After filtered through a mixed cellulose ester membrane filter (0.45 μm, ADVANTEC, Tokyo, Japan), the membranes were cut into pieces. The viruses were eluted from the membranes, using 10 ml of a 3 % beef extract solution (pH = 9), followed by ultrasonication for 5 min. The eluted solution was centrifuged at 3000 × *g* for 30 min, and the supernatant was filtered through a 0.2 μm filter (PALL, Ann Arbor, MI, USA) prior to RNA extraction.

### Quantification of rotavirus

High Pure Viral Nucleic Acid Large Volume Kit (Roche, Indiana, USA) was used to extract viral RNA from 1 ml of concentrated sewage. Because the samples all tested negative for poliovirus type 1 Sabin strain, concentrated sewage samples were seeded with a known amount of Sabin 1 as a control to monitor the efficiency of RNA extraction and reverse transcription-qPCR (RT-qPCR). RT-qPCR for rotavirus was carried out by QuantiTect Probe RT-PCR Kit (Qiagen, Hilden, Germany). Briefly, the reaction mixture (25 μl) consisted of 12.5 μl 2× QuantiTect Probe RT-PCR Master Mix, 0.4 μmol l^−1^ final concentration of each primer, 0.2 μmol l^−1^ final concentration of each probe, 0.25 μl QuantiTect RT Mix and 10 μl template RNA. The primers and probe towards NSP3 gene were previously described by Freeman et al. [15]. Each sample was measured in triplicate. All qPCR reactions were carried out in an ABI 7500 Real-Time System (Applied Biosystems, Foster City, California, USA). To determine the copy numbers of rotavirus genome, a standard curve was conducted by 10-fold serial dilution (10^1^ ~ 10^9^) of plasmid DNA containing the target gene. All of the concentrations of rotavirus presented were transformed to genome copies per litre (GC/L) according to the volumes used for each step of the procedure. To avoid cross contamination, molecular procedures were performed in different separated rooms and a negative control was included in every qPCR run.

### Conventional RT-PCR

In rotavirus-positive sewage samples confirmed by RT-qPCR, conventional RT-PCR was performed with two improved primer sets by using the Access RT-PCR System (Promega, Fitchburg, WI, USA). Primer set VP4F.142S (TAT GCI CCW GTI AMT TGG) and VP4R.805A (ATT GCA TTT CYT TCC AYA AYG) was used for amplification of a 664-nucleotide (nt) VP4 sequence according to nt position 142–805 of strain Human-tc/USA/Wa/1974 segment 4 (JX406750). Primer set VP7F.49S (ATG TAT GGT ATT GAA TAT ACC) and VP7R.932A (ACT TGC CAC CAT YTY TTC C) was used for amplification of a 884-nt VP7 sequence according to nt position 49–932 of strain Human-tc/USA/Wa/1974 segment 9 (JX406755).

### Cloning and sequencing

RT-PCR products were analyzed by electrophoresis with 1.5 % agarose gels. All positive products were gel-purified by QIAquick gel extraction kit (Qiagen, Valencia, CA, USA) and ligated into the pGEM®-T Easy vector (Promega). By shock method, the ligation products were transformed into competent *Escherichia coli* JM109 cells. For each transformation reaction, ten positive clones were selected after blue and white screening. Then, the plasmid was extracted and sequenced with a BigDye Terminator v3.1 Cycle Sequencing Kit (Applied Biosystems, Foster City, CA, USA), on an ABI 3130 genetic analyzer (Applied Biosystems). Molecular typing was carried out by using BLAST.

### Sequences analysis

Nucleotide sequence alignments were carried out by BioEdit 7.1.3.0 [16]. Recombination event was analyzed by RDP software package [17]. Mega 5.0 was used to construct the phylogenetic tree by using the neighbor-joining method [18]. Bootstrapping test was performed with 1000 duplicates. Nucleotide sequences used in phylogenetic analysis from this study were deposited in GenBank under accession numbers KU173953-KU174110.

## Results

### Concentration of rotavirus

**Fig. 1:**
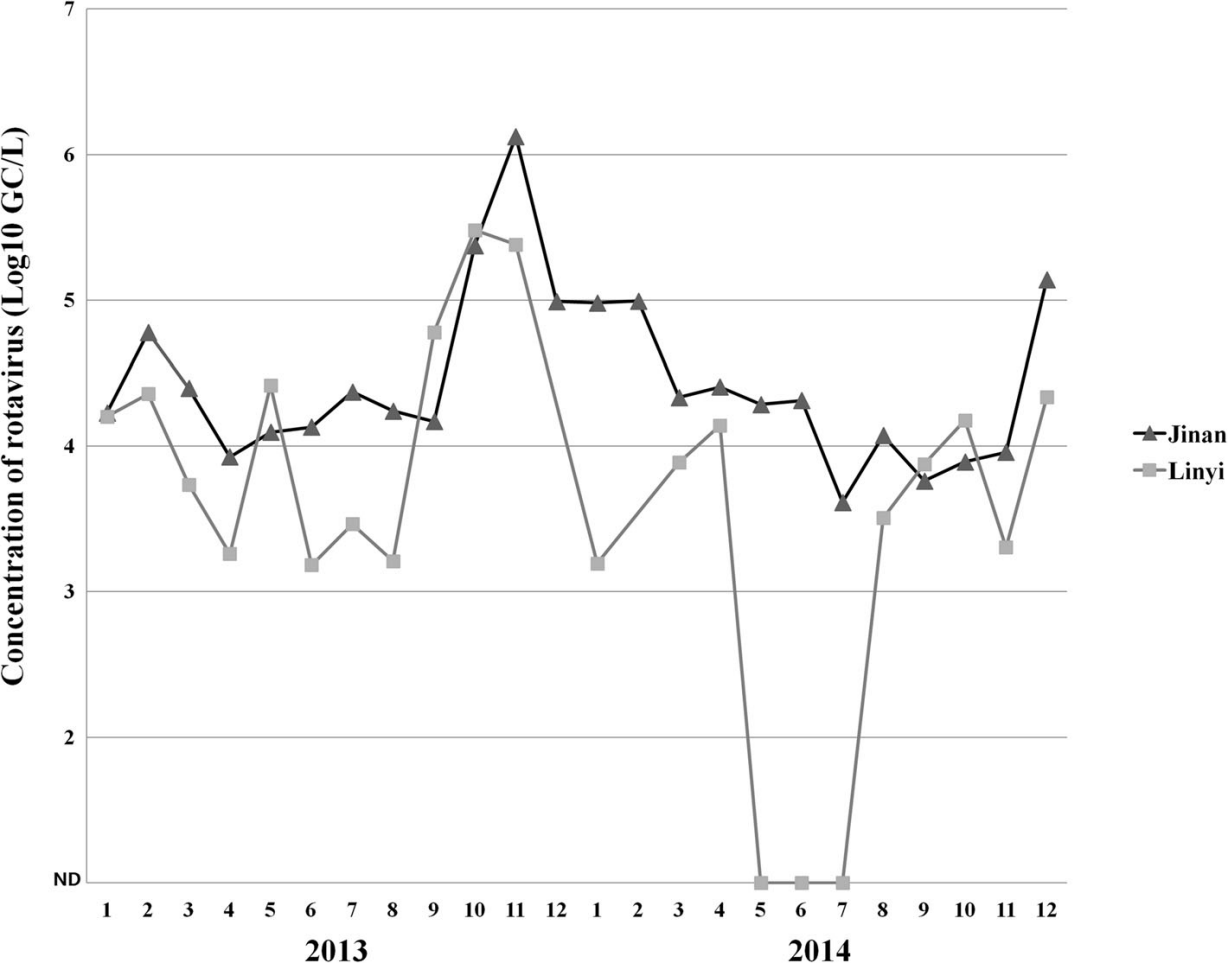
Concentrations of rotavirus in raw sewage samples from Jinan and Linyi. The plots show monthly the mean values (Log10 GC/L). Sewage samples in December 2013 and February 2014 were not available in Linyi. ND means not detected

During the 2-year period, a total of 46 raw sewage samples (23 in Jinan and 23 in Linyi) were collected. After RT-qPCR detection, rotavirus was positive in 43 samples (93.5 %, 43/46), 24 (100 %, 24/24) in Jinan and 19 (86.4 %, 19/22) in Linyi, respectively. The RNA extraction and RT-qPCR efficiency of Sabin 1 was 74.5 ± 11.3 % (*n* = 46), and the efficiency for each sample was used to estimate the actual concentration of rotavirus in the sample. Figure 1 shows the monthly concentration of rotavirus in raw sewage. In Jinan, the concentrations of rotavirus ranged from 4.1 × 10^3^ to 1.3 × 10^6^ GC/L, and were relatively high in November 2013 and December 2014. In Linyi, the concentrations of rotavirus ranged from 1.5 × 10^3^ to 3.0 × 10^5^ GC/L, and were relatively high in October 2013.

### Distribution of rotavirus genotypes

**Fig. 2:**
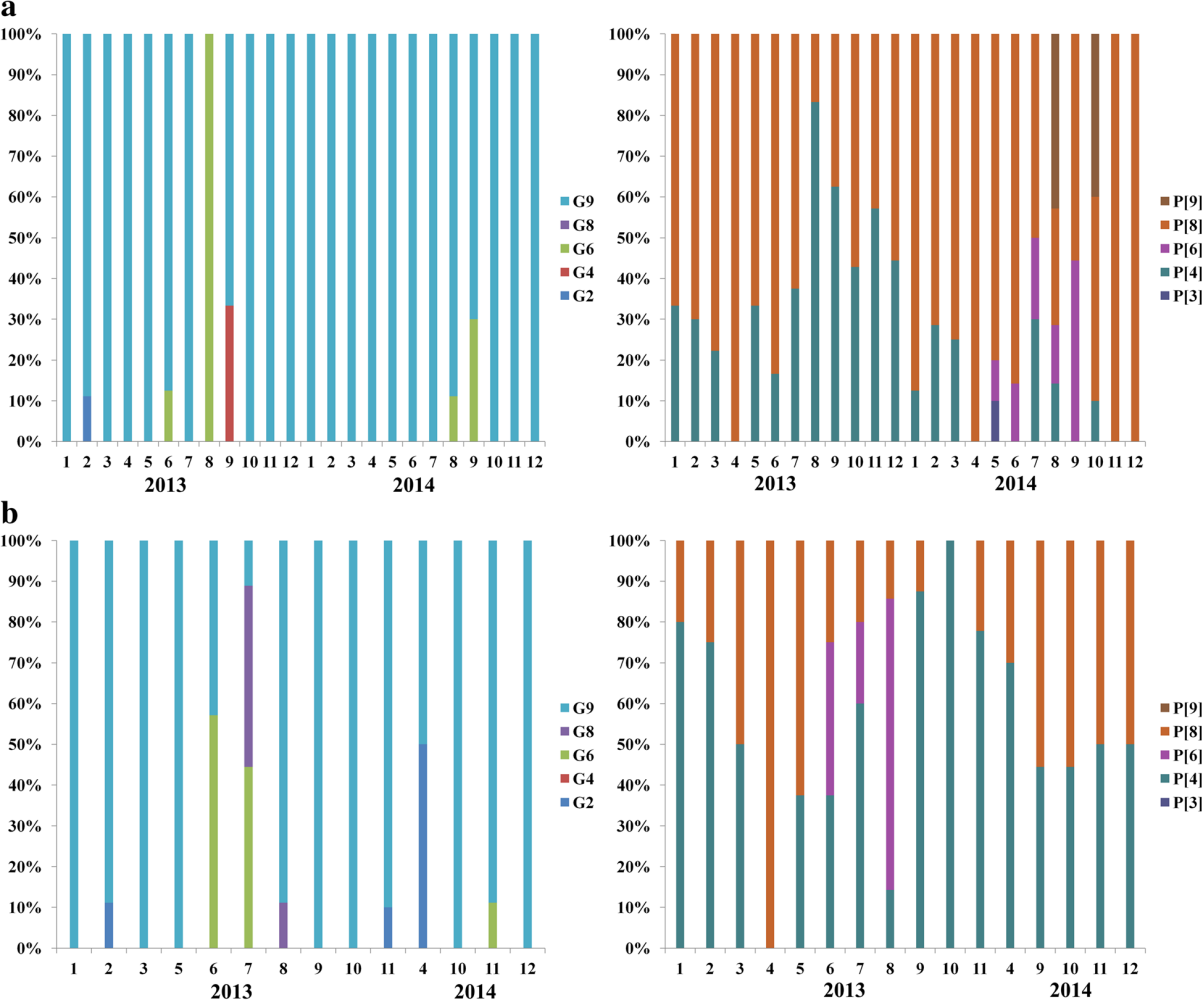
Relative proportions of genotypes in detected samples. The column graphs show the month-by-month relative proportions of G- and P-genotypes in raw sewage samples from Jinan (**a**) and Linyi (**b**). Sewage samples were not available in December, 2013 and February, 2014 of Liny, and rotavirus was not detected in May, June and July, 2014 of Linyi. No sequence was obtained in some months of Linyi

By conventional RT-PCR, rotavirus was detected in all rotavirus-positive samples confirmed by RT-qPCR. After cloning and sequencing, 318 G-genotypes sequences (200 in Jinan and 118 in Linyi) and 318 P-genotypes sequences (200 in Jinan and 118 in Linyi) were obtained, belonging to 5 G- (G9, G6, G8, G4 and G2) and 5 P-genotypes (P[8], P[4], P[3], P[6] and P[9]), respectively. G9 was the most common G-genotype, accounting for 91.8 % (292/318). Other G-genotypes were all detected at a low prevalence. P[8] and P[4] were two most common P-genotypes, accounting for 56.0 % (178/318) and 35.8 % (114/318). P[9] and P[3] were only found in Jinan, with the detection rate of 3.5 % (7/200) and 0.5 % (1/200). Figure 2 shows the month-by-month relative proportions of genotypes.

### Sequences analysis

Homologous comparison was performed in all obtained sequences from Jinan and Linyi. To reduce workload, Shandong G9, P[8] and P[4] sequences displaying more than 99.5 % nucleotide identities within these genotypes have been deleted. Phylogenetic analysis was conducted between the remaining sequences and reference strains selected randomly from GenBank database.

**Fig. 3:**
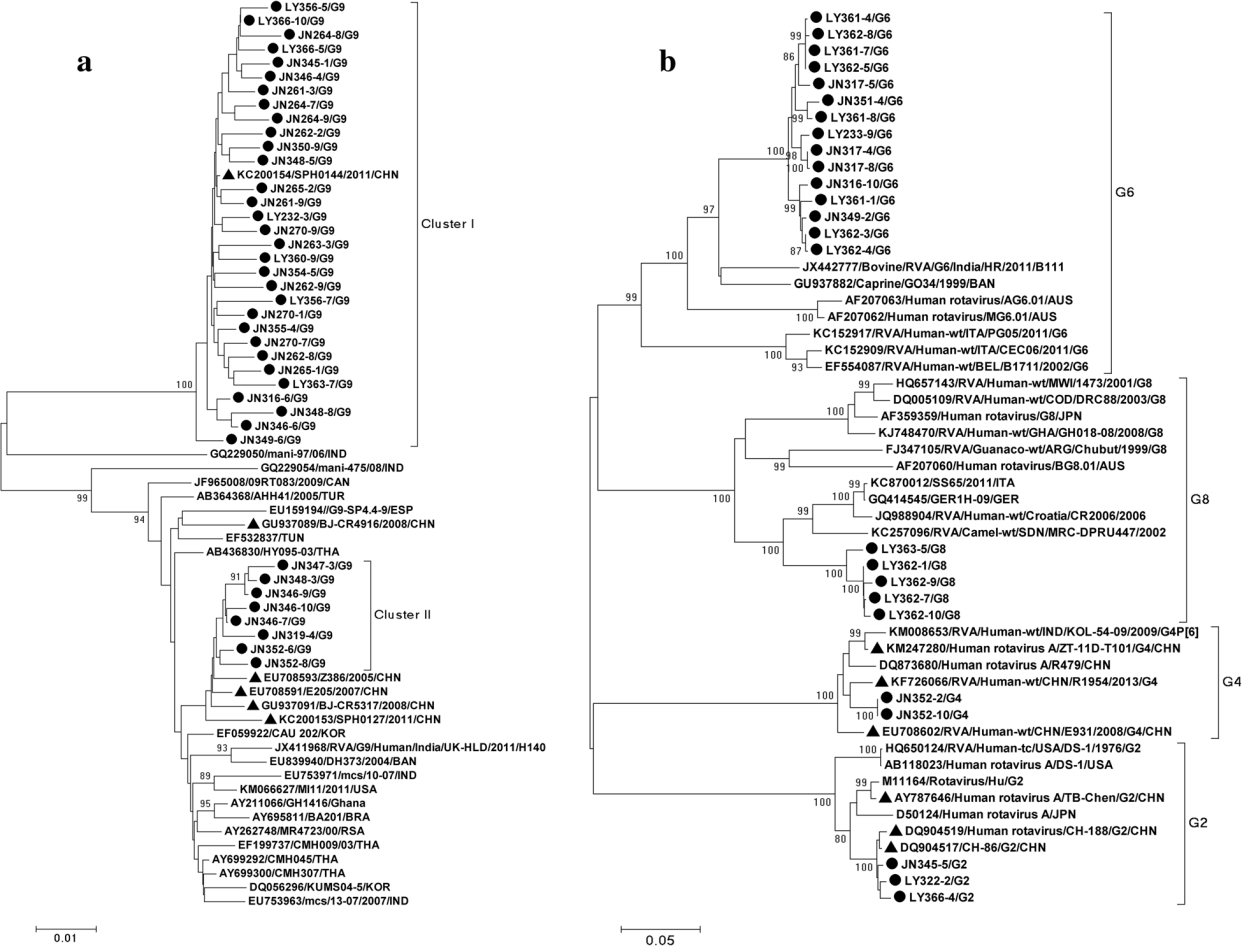
Phylogenetic relationship of G9 sequences (**a**), G2, G4, G6 and G8 sequences (**b**). The phylogenetic trees were constructed based on 884-nt RVA VP7 sequence according to nt position 49–932 of strain Human-tc/USA/Wa/1974 segment 9 (JX406755). ● and ▲ indicate sequences from sewage sample and reference strains from China, respectively

Phylogenetic relationships of G-genotypes were illustrated in Fig. 3. Shandong G9 sequences can be divided into two clusters, and cluster II only contained Jinan sequences. Two Chinese reference strains from cases in Shanghai (KC200154) and Wuhan (EU708591) displayed close relationship with sequences in cluster I (98.8–99.5 %) and cluster II (98.6–99.2 %), respectively. Another two Chinese reference strains (DQ904517 and KF726066) showed high similarity with Shandong G2 (98.8–99.3 %) and G4 (96.9 %) sequences, respectively. Shandong G6 sequences were found to be more closely related to reference strains from animals (JX442777 and GU937882) than classical human G6 sequences (89.9–91.1 % vs. 81.1–86.4 %). A human G8 reference strain from Germany (GQ414545) and an animal G8 reference strain from Sudan (KC257096) presented similar nucleotide identity to Shandong G8 sequences (89.7–90.2 % vs. 90.6–91.1 %).

**Fig. 4:**
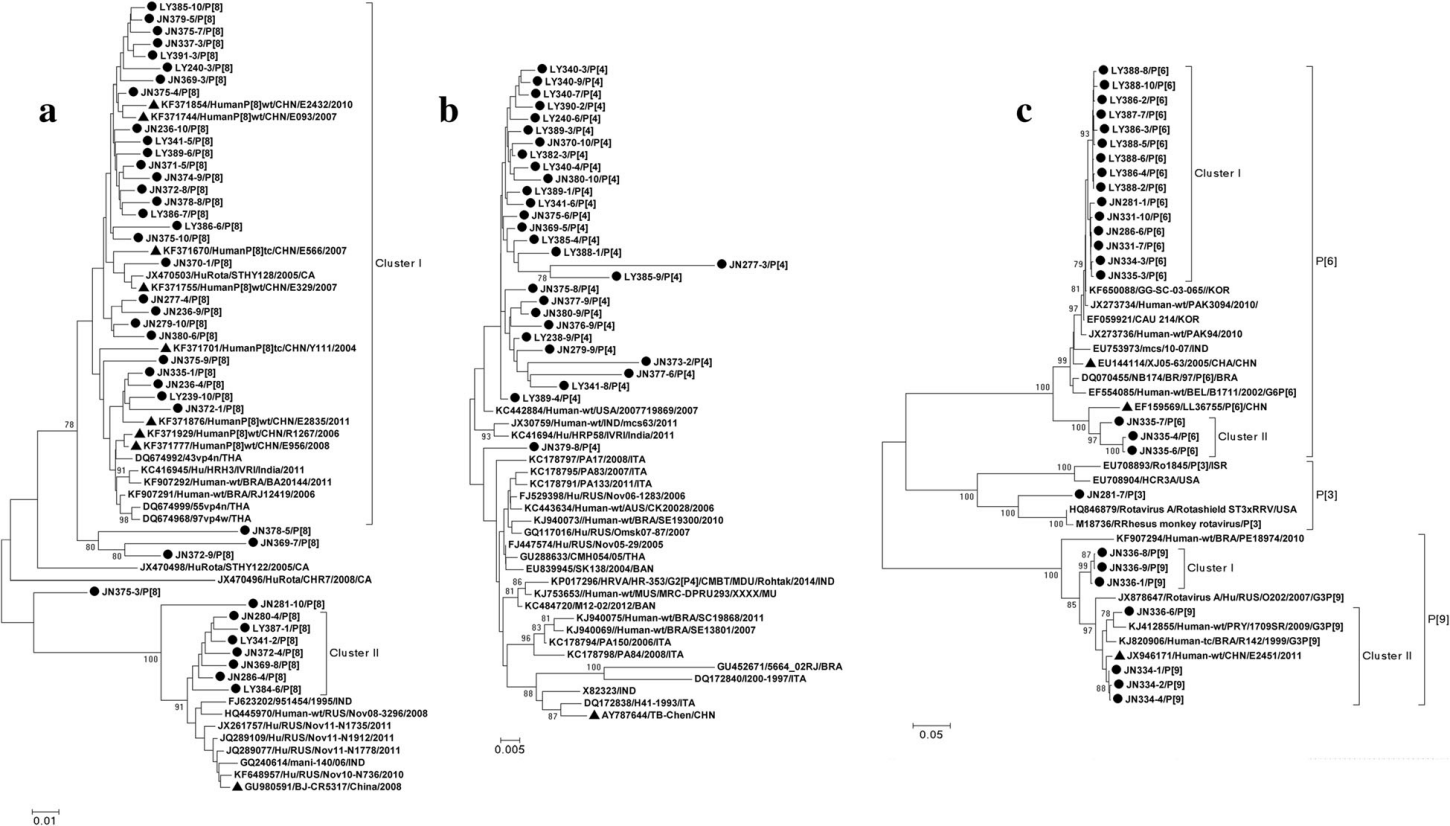
Phylogenetic relationship of P[8] sequences (**a**), P[4] sequences (**b**), P[3], P[6] and P[9] sequences (**c**). The phylogenetic trees were constructed based on 664-nt RVA VP4 sequence according to nt position 142–805 of strain Human-tc/USA/Wa/1974 segment 4 (JX406750). ● and ▲ indicate sequences from sewage sample and reference strains from China, respectively

Phylogenetic relationships of P-genotypes were shown in Fig. 4. P[4] sequences were located at one main phylogenetic branch except one sequence in Jinan (JN379-8), and great genetic difference was observed in this branch (91.5–99.5 % identity). Within selected P[8] sequences, two clusters were recognized after excluding sequences JN378-5, JN369-7, JN372-9 and JN375-3. Additionally, Chinese reference strains from Wuhan showed close relationship with Shandong P[8] sequences in the major cluster (94.7–99.5 % identity). Shandong P[6] and P[9] sequences can also be separated into two clusters. P[3] sequence in this study displayed low similarity with human [P3] reference strain (76.1 %), but high similarity with rhesus rotavirus reference strains (87.2–87.8 %).

### Recombination

**Fig. 5:**
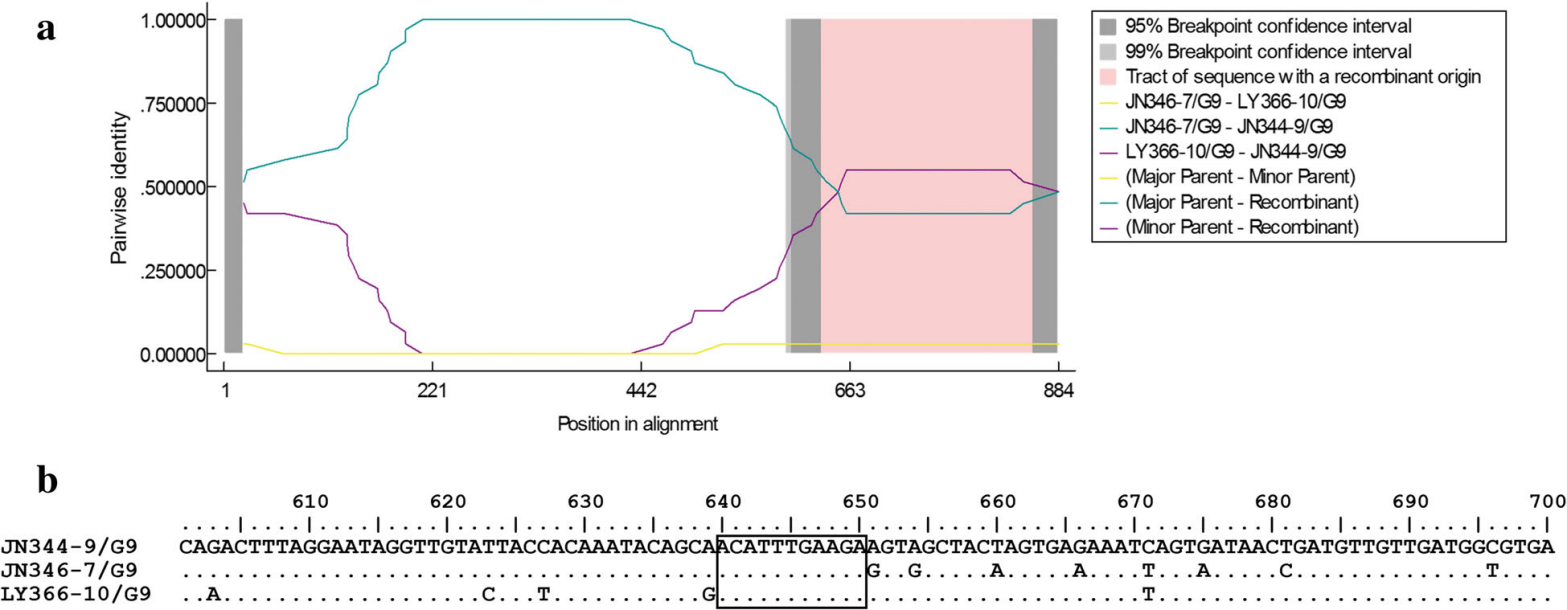
Intragenic recombination event in one G9 sequence (JN344-9) from sewage. **a** Pairwise identity plot by RDP software version 4.0; **b** Sequence in the *box* indicates crossover sites

After RDP analysis and the alignment of 884-nt G9 sequences, an intragenic recombination event was observed in one G9 sequence (JN344-9) (Fig. 5). The crossover sites ranged from position 688 to 698 according to the sequence of strain Human-tc/USA/Wa/1974 segment 9 (JX406755).

## Discussion

Environmental sewage surveillance has been successfully conducted to obtain molecular characterizations of human enteric viruses in some regions [19–21]. In this study, this novel surveillance method was performed to monitor the circulation of rotavirus in Jinan and Linyi of eastern China. During the 2-year period of monitoring, a large number of rotavirus sequences were obtained and revealed the molecular characterizations of rotaviruses circulating in the local population.

At present, various detection rates of rotavirus in urban sewage have been reported [22, 23]. In this study, rotavirus was detected at a high rate (93.5 %) both by conventional RT-PCR and RT-qPCR, reflecting the high prevalence of rotavirus in the local population. Sewage surveillance has been discussed to be a useful tool to evaluate the circulation of rotavirus after rotavirus vaccination, and low detection rates of rotavirus in sewage were observed in some regions with universal rotavirus vaccination [24, 25]. In view of the high detection rate of rotavirus in this study, rotavirus vaccination should be included in the National Immunization Program in China. Rotavirus infection is seasonal, and shows a peak in cold seasons [26]. In this study, RT-qPCR results showed that the concentrations of rotavirus in raw sewage tended to peak in autumn and winter months in Jinan, revealing that RT-qPCR could be an effective approach to exploring the seasonal profile of rotavirus in the population. In Linyi, no apparent seasonality was observed both by conventional RT-PCR and RT-qPCR. This phenomenon might indicate that asymptomatic rotavirus infection was common in this city [5, 23]. The infectious dose of rotavirus is low (<100 virus particles) [27]. The relative abundance of rotavirus in sewage in this study shows that the public health will be threatened if drinking water or food is contaminated by sewage, although the actual level of rotavirus with infectivity may be overestimated.

Currently, G1-G4, G9, P[4], P[6] and P[8] are the prevalent genotypes worldwide [28]. In this study, G9 and P[8] were recognized predominantly, showing that they were the dominant genotypes in this region. This data also suggested that G9P[8] might be the most prevalent G-P genotypes combination circulating in the local population, as previously described in other regions of China [29, 30]. As two most frequently detected G-genotypes of China in the past decades, G1 and G3 were not recognized in this study, and the detection rates of common G2 and G4 were also low, which may indicate that common G1-G4 were currently circulating at a very low prevalence in the two cities.

G9 infections are globally increasing at the exponential growth since 1993, and G9 have recently been the significant genotype in some regions. In this study, G9 was also observed as the predominant G-genotype and two main phylogenetic clusters were identified. The minor cluster was only recognized in Jinan in 2013, showing its local circulation in Jinan. The major cluster was continuously observed in Jinan and Linyi from 2013 to 2014, revealing that it was the main G9 transmission lineage in the two cities and the inter-city spread may exist. Interestingly, an intragenic recombination event between two G9 lineages was observed in this study. Intragenic recombination in rotavirus is rare until increasing reports recently [31, 32]. To our knowledge, this is the first report of intragenic recombination in G9 rotaviruses. Mixed infections between different variants belonging to the same genotype were discussed as the cause of intragenic recombination [33]. Thus, co-circulation of two G9 transmission lineages in Jinan in 2013 might lead to the occurrence of this recombination event.

G6 and G8 strains were reported to be of animal origin [34, 35]. In this study, some G6 and G8 sequences were obtained. By BLAST, we found that these sequences showed the highest identities to animal sequences (although 10 % divergence is quite different), and no human rotavirus sequences showed higher identities to them. We cannot confirm that these sequences were from humans or animals. They also might be the novel variants circulating in the local population. All of these need to be confirmed by case-based surveillance in the future.

The genetic differences within P[4], P[8], P[6] and P[9] sequences were high, and more than one cluster was recognized, suggesting the co-circulation of multiple transmission lineages in these genotypes. P[3] sequence in this study showed the highest identity to the rhesus rotavirus P[3] strain (RRV strain, M18736). RRV is the recipient virus in human-rhesus reassortant vaccine [36], but the detection of RRV-like P-genotype is rare so far. This is the first recognition of it in sewage. Nevertheless, the meaning of this finding is unknown because there is no evidence to support the epidemiological relevance of RRV P[3] in humans [37]. Further research on rare RRV P[3] is needed.

## Conclusions

In conclusion, this study shows that the concentration of rotavirus in raw sewage was high, and multiple rotavirus transmission lineages continuously co-circulated in Jinan and Linyi, eastern China. Additionally, this study proves that environmental surveillance is an ideal measure to obtain epidemiological data on circulating viruses in a given population.

## Abbreviations

dsRNA: Double-stranded RNA
GC: Genome copies
LLR: Lanzhou lamb rotavirus
NSPs: Nonstructural proteins
RT-qPCR: Reverse transcription-quantitative PCR
RVA: Rotavirus group A
VPs: Structural proteins
WWTPs: Wastewater treatment plants
